# Supplementary material for: Effect of chemical modifications of tannins on their antimicrobial and antibiofilm effect against Gram-negative and Gram-positive bacteria
Source: Front Microbiol. 2023 Jan 6;13:987164. doi: 10.3389/fmicb.2022.987164 (PMC9853077; doi:10.3389/fmicb.2022.987164)
Supplement: Supplementary file 1 [file Table_1.DOCX]

**TABLE S1.** BIC_50_ (compound concentration in mg/l required to inhibit the biofilm formation by 50%) of the different assayed tannins.

| **Unmodified Tannins** | **Salmonella Typhimurium** | **95% CI** | **Pseudomonas aeruginosa** | **95% CI** | **Escherichia coli** | **95% CI** | **Staphylococcus aureus** | **95% CI** |
| --- | --- | --- | --- | --- | --- | --- | --- | --- |
| Vv-20 | 25.5 | 22.5 to 28.8 | 36.5 | 9.6 to 138.6 | > 600.0 | ǂ | > 600.0 | ǂ |
| Vv | > 600.0 | ǂ | 7.8 | 1.5 to 39.9 | > 600.0 | ǂ | > 600.0 | ǂ |
| Am | ~ 18.7 | ǂ | ~ 152.7 | ǂ | > 600.0 | ǂ | > 600.0 | ǂ |
| Ta-01 | ~ 523.8 | ǂ | 25.6 | 19.7 to 33.4 | > 600.0 | ǂ | ~ 145.4 | ǂ |
| Ta-04 | 9.8 | 0.5 to 191.0 | ~ 545.8 | ǂ | 24.7 | 6.8 to 89.2 | 8.1 | 2.5 to 25.7 |
| **Blank Reaction Tannins** | **Salmonella Typhimurium** | **95% CI** | **Pseudomonas aeruginosa** | **95% CI** | **Escherichia coli** | **95% CI** | **Staphylococcus aureus** | **95% CI** |
| Vv-20-Blank-W | 159.5 | 140.6 to 180.8 | > 600.0 | ǂ | > 600.0 | ǂ | > 600.0 | ǂ |
| Vv-Blank-W | 357.4 | 24.6 to 5192.0 | > 600.0 | ǂ | > 600.0 | ǂ | > 600.0 | ǂ |
| Am-Blank-W | 51.7 | 39.5 to 67.8 | ~ 296.4 | ǂ | > 600.0 | ǂ | > 600.0 | ǂ |
| Vv-20-Blank-D | 26.2 | 21.8 to 31.4 | ~ 0.2 | ǂ | > 600.0 | ǂ | > 600.0 | ǂ |
| Vv-Blank-D | 11.5 | 4.4 to 29.7 | 22.2 | 12.2 to 40.2 | > 600.0 | ǂ | > 600.0 | ǂ |
| Am-Blank-D | 19.3 | 17.9 to 20.7 | 166.4 | 135.8 to 203.9 | > 600.0 | ǂ | > 600.0 | ǂ |
| Ta-01-Blank-D | ~ 2.7 | ǂ | 6 | 0.4 to 86.7 | 10.7 | 6.1 to 18.6 | 117.5 | 43.1 to 320.6 |
| Ta-04-Blank-D | 124.2 | 41.36 to 373.1 | > 600.0 | ǂ | > 600.0 | ǂ | ~ 35.8 | ǂ |
| **Modified Tannins** | **Salmonella Typhimurium** | **95% CI** | **Pseudomonas aeruginosa** | **95% CI** | **Escherichia coli** | **95% CI** | **Staphylococcus aureus** | **95% CI** |
| Vv-20-C_3_NMe_3_Cl-0.1 | > 600.0 | ǂ | > 600.0 | ǂ | > 600.0 | ǂ | > 600.0 | ǂ |
| Vv- C_3_NMe_3_Cl-0.1 | > 600.0 | ǂ | > 600.0 | ǂ | > 600.0 | ǂ | > 600.0 | ǂ |
| Am- C_3_NMe_3_Cl-0.1 | > 600.0 | ǂ | > 600.0 | ǂ | > 600.0 | ǂ | > 600.0 | ǂ |
| Ta-01- C_3_NMe_3_Cl-0.1 | > 600.0 | ǂ | > 600.0 | ǂ | > 600.0 | ǂ | > 600.0 | ǂ |
| Ta-04- C_3_NMe_3_Cl-0.1 | > 600.0 | ǂ | > 600.0 | ǂ | > 600.0 | ǂ | > 600.0 | ǂ |
| Vv-20- C_3_NMe_3_Cl-0.1 | > 600.0 | ǂ | > 600.0 | ǂ | > 600.0 | ǂ | > 600.0 | ǂ |
| Vv-20-C_3_NMe_3_Cl-0.5 | ~ 8.8 | ǂ | 26.8 | 19.8 to 36.1 | 21.7 | 14.6 to 32.0 | > 600.0 | ǂ |
| Vv- C_3_NMe_3_Cl-0.5 | 12.7 | 8.7 to 18.6 | 28.3 | 6.0 to 132.9 | 7.7 | 0.1 to 1096.0 | > 600.0 | ǂ |
| Am- C_3_NMe_3_Cl-0.5 | > 600.0 | ǂ | 6.3 | 2.5 to 15.9 | 21.4 | 16.3 to 28.0 | > 600.0 | ǂ |
| Ta-01- C_3_NMe_3_Cl-0.5 | ~ 9.3 | ǂ | 21.6 | 10.0 to 46.8 | ~ 2.6 | ǂ | > 600.0 | ǂ |
| Ta-04- C_3_NMe_3_Cl-0.5 | > 600.0 | ǂ | > 600.0 | ǂ | > 600.0 | ǂ | ~ 35.7 | ǂ |
| Vv-C_3_COOH-0.1 | > 600.0 | ǂ | > 600.0 | ǂ | > 600.0 | ǂ | > 600.0 | ǂ |
| Am- C_3_COOH-0.1 | ~ 299.2 | ǂ | > 600.0 | ǂ | > 600.0 | ǂ | > 600.0 | ǂ |
| Ta-01- C_3_COOH-0.1 | ~ 9.1 | ǂ | 75.43 | 66.8 to 85.1 | > 600.0 | ǂ | 238.2 | 94.2 to 602.3 |
| Ta-04- C_3_COOH-0.1 | 64.7 | 55.0 to 76.1 | > 600 | ǂ | 79.3 | 54.6 to 115.3 | > 600.0 | ǂ |
| Vv-20-C_3_COOH-0.5 | > 600.0 | ǂ | > 600.0 | ǂ | > 600.0 | ǂ | > 600.0 | ǂ |
| Vv-C_3_COOH-0.5 | 10.02 | 2.8 to 35.8 | ~ 0.6 | ǂ | > 600.0 | ǂ | ~ 153.7 | ǂ |
| Am-C_3_COOH-0.5 | > 600.0 | ǂ | > 600.0 | ǂ | > 600.0 | ǂ | > 600.0 | ǂ |
| Ta-01-C_3_COOH-0.5 | ~ 4.6 | ǂ | 24.4 | 15.6 to 38.0 | > 600.0 | ǂ | ~ 126.3 | ǂ |
| Ta-04-C_3_COOH-0.5 | 13.1 | 0.1 to 1952.0 | ~ 323.3 | ǂ | > 600.0 | ǂ | 13.6 | 11.2 to 16.6 |
| Vv-PEG-0.05 | ~ 19.1 | ǂ | < 0.1 | ǂ | > 600.0 | ǂ | ~ 8.8 | ǂ |
| Am-PEG-0.05 | > 600.0 | ǂ | ~ 299.9 | ǂ | > 600.0 | ǂ | > 600.0 | ǂ |
| Ta-01-PEG-0.05 | ~ 9. 0 | ǂ | 102.8 | 56.7 to 186.2 | > 600.0 | ǂ | > 600.0 | ǂ |

The ~ next to a value indicate those BIC_50_ values could that not be accurately calculated due to the steepness of the curve

ǂ: Not possible to calculate 95% CI due to steepness of the curve
