# Supplementary material for: Effect of chemical modifications of tannins on their antimicrobial and antibiofilm effect against Gram-negative and Gram-positive bacteria
Source: Front Microbiol. 2023 Jan 6;13:987164. doi: 10.3389/fmicb.2022.987164 (PMC9853077; doi:10.3389/fmicb.2022.987164)
Supplement: Supplementary file 2 [file Table_2.DOCX]

**TABLE S2.** IC_50_ (the compound concentration in mg/l required to inhibit the bacterial growth by 50%) of the different assayed tannins.

| **Unmodified Tannins** | ***Salmonella* Typhimurium** | **95% CI** | ***Pseudomonas aeruginosa*** | **95% CI** | ***Escherichia coli*** | **95% CI** | ***Staphylococcus aureus*** | **95% CI** |
| --- | --- | --- | --- | --- | --- | --- | --- | --- |
| Vv-20 | > 600.0 | ǂ | > 600.0 | ǂ | > 600.0 | ǂ | > 600.0 | ǂ |
| Vv | > 600.0 | ǂ | 232.5 | 203.4 to 265.8 | > 600.0 | ǂ | > 600.0 | ǂ |
| Am | > 600.0 | ǂ | > 600.0 | ǂ | > 600.0 | ǂ | > 600.0 | ǂ |
| Ta-01 | 274.3 | 201.3 to 373.9 | > 600.0 | ǂ | 459.2 | 297.8 to 708.1 | > 600.0 | ǂ |
| Ta-04 | > 600.0 | ǂ | 408.3 | 325.6 to 511.9 | > 600.0 | ǂ | > 600.0 | ǂ |
| **Blank Reaction Tannins** | ***Salmonella* Typhimurium** | **95% CI** | ***Pseudomonas aeruginosa*** | **95% CI** | ***Escherichia coli*** | **95% CI** | ***Staphylococcus aureus*** | **95% CI** |
| Vv-20-Blank-W | > 600.0 | ǂ | > 600.0 | ǂ | > 600.0 | ǂ | > 600.0 | ǂ |
| Vv-Blank-W | > 600.0 | ǂ | > 600.0 | ǂ | > 600.0 | ǂ | > 600.0 | ǂ |
| Am-Blank-W | > 600.0 | ǂ | > 600.0 | ǂ | > 600.0 | ǂ | > 600.0 | ǂ |
| Vv-20-Blank-D | > 600.0 | ǂ | > 600.0 | ǂ | > 600.0 | ǂ | > 600.0 | ǂ |
| Vv-Blank-D | > 600.0 | ǂ | > 600.0 | ǂ | 352.3 | 297.4 to 417.3 | > 600.0 | ǂ |
| Am-Blank-D | > 600.0 | ǂ | 43.6 | 0.0 to 509449930585.0 | > 600.0 | ǂ | > 600.0 | ǂ |
| Ta-01-Blank-D | 218.9 | 81.2 to 590.5 | > 600.0 | ǂ | 159.3 | 118.6 to 213.8 | > 600.0 | ǂ |
| Ta-04-Blank-D | > 600.0 | ǂ | 217.5 | 103.3 to 457.6 | > 600.0 | ǂ | > 600.0 | ǂ |
| **Modified Tannins** | ***Salmonella* Typhimurium** | **95% CI** | ***Pseudomonas aeruginosa*** | **95% CI** | ***Escherichia coli*** | **95% CI** | ***Staphylococcus aureus*** | **95% CI** |
| Vv-20-C_3_NMe_3_Cl-0.1 | > 600.0 | ǂ | > 600.0 | ǂ | > 600.0 | ǂ | > 600.0 | ǂ |
| Vv-C_3_NMe_3_Cl-0.1 | > 600.0 | ǂ | > 600.0 | ǂ | > 600.0 | ǂ | > 600.0 | ǂ |
| Am-C_3_NMe_3_Cl-0.1 | > 600.0 | ǂ | > 600.0 | ǂ | > 600.0 | ǂ | > 600.0 | ǂ |
| Ta-01-C_3_NMe_3_Cl-0.1 | > 600.0 | ǂ | > 600.0 | ǂ | > 600.0 | ǂ | > 600.0 | ǂ |
| Ta-04-C_3_NMe_3_Cl-0.1 | > 600.0 | ǂ | 221.7 | 175.4 to 280.2 | > 600.0 | ǂ | > 600.0 | ǂ |
| Vv-20-C_3_NMe_3_Cl-0.5 | > 600.0 | ǂ | > 600.0 | ǂ | > 600.0 | ǂ | > 600.0 | ǂ |
| Vv-C_3_NMe_3_Cl-0.5 | > 600.0 | ǂ | > 600.0 | ǂ | > 600.0 | ǂ | > 600.0 | ǂ |
| Am-C_3_NMe_3_Cl-0.5 | > 600.0 | ǂ | > 600.0 | ǂ | > 600.0 | ǂ | > 600.0 | ǂ |
| Ta-01-C_3_NMe_3_Cl-0.5 | > 600.0 | ǂ | > 600.0 | ǂ | > 600.0 | ǂ | > 600.0 | ǂ |
| Ta-04-C_3_NMe_3_Cl-0.5 | > 600.0 | ǂ | > 600.0 | ǂ | > 600.0 | ǂ | 109.1 | ǂ |
| Vv-20-C_3_COOH-0.1 | > 600.0 | ǂ | > 600.0 | ǂ | > 600.0 | ǂ | > 600.0 | ǂ |
| Vv-C_3_COOH-0.1 | > 600.0 | ǂ | > 600.0 | ǂ | > 600.0 | ǂ | > 600.0 | ǂ |
| Am-C_3_COOH-0.1 | > 600.0 | ǂ | > 600.0 | ǂ | > 600.0 | ǂ | > 600.0 | ǂ |
| Ta-01-C_3_COOH-0.1 | > 600.0 | ǂ | > 600.0 | ǂ | 113.2 | 52.6 to 243.9 | > 600.0 | ǂ |
| Ta-04-C_3_COOH-0.1 | > 600.0 | ǂ | 169.3 | 133.7 to 214.4 | 179 | 121.2 to 264.3 | > 600.0 | ǂ |
| Vv-20-C_3_COOH-0.5 | > 600.0 | ǂ | > 600.0 | ǂ | > 600.0 | ǂ | > 600.0 | ǂ |
| Vv-C_3_COOH-0.5 | > 600.0 | ǂ | > 600.0 | ǂ | > 600.0 | ǂ | > 600.0 | ǂ |
| Am-C_3_COOH-0.5 | > 600.0 | ǂ | > 600.0 | ǂ | > 600.0 | ǂ | > 600.0 | ǂ |
| Ta-01-C_3_COOH-0.5 | 282.9 | 71.4 to 1121.0 | > 600.0 | ǂ | > 600.0 | ǂ | > 600.0 | ǂ |
| Ta-04-C_3_COOH-0.5 | > 600.0 | ǂ | > 600.0 | ǂ | > 600.0 | ǂ | > 600.0 | ǂ |
| Vv-PEG -0.05 | > 600.0 | ǂ | > 600.0 | ǂ | ~ 149.7 | ǂ | 155.2 | 37.6 to 641.4 |
| Am-PEG-0.05 | > 600.0 | ǂ | > 600.0 | ǂ | > 600.0 | ǂ | > 600.0 | ǂ |
| Ta-01-PEG-0.05 | > 600.0 | ǂ | > 600.0 | ǂ | > 600.0 | ǂ | > 600.0 | ǂ |

ǂ: Not possible to calculate 95% CI due to steepness of the curve
