# Supplementary material for: Effect of chemical modifications of tannins on their antimicrobial and antibiofilm effect against Gram-negative and Gram-positive bacteria
Source: Front Microbiol. 2023 Jan 6;13:987164. doi: 10.3389/fmicb.2022.987164 (PMC9853077; doi:10.3389/fmicb.2022.987164)
Supplement: Supplementary file 4 [file Image_2.PDF]

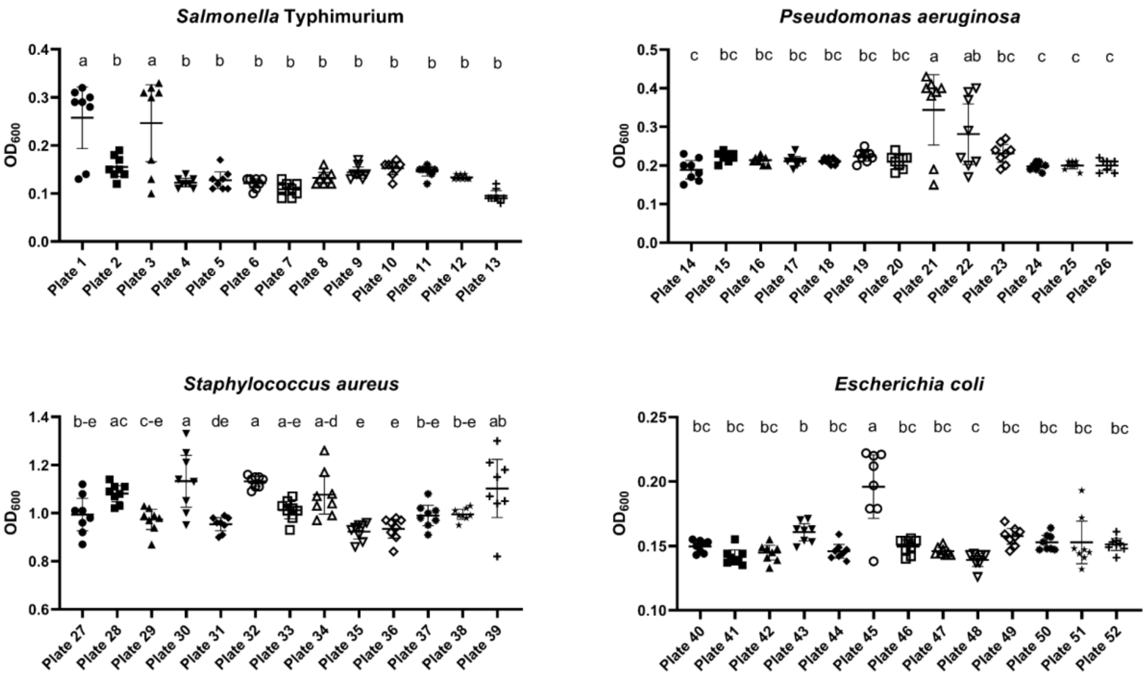

FIG S2. Variability of the planktonic (OD_{600}) positive controls per plate per each of the assayed bacteria in the exploratory screening for activity of tannins against planktonic bacteria. To measure the effect of the plate in assay, a one-way ANOVA with Welch's correction for non-similar variances and Tukey-Kramer HSD post-hoc test was performed. The letters represent groups of plates that are not significantly different from each other, and the error bars represent 95% confidence interval.
