# Supplementary material for: Effect of chemical modifications of tannins on their antimicrobial and antibiofilm effect against Gram-negative and Gram-positive bacteria
Source: Front Microbiol. 2023 Jan 6;13:987164. doi: 10.3389/fmicb.2022.987164 (PMC9853077; doi:10.3389/fmicb.2022.987164)
Supplement: Supplementary file 5 [file Image_3.PDF]

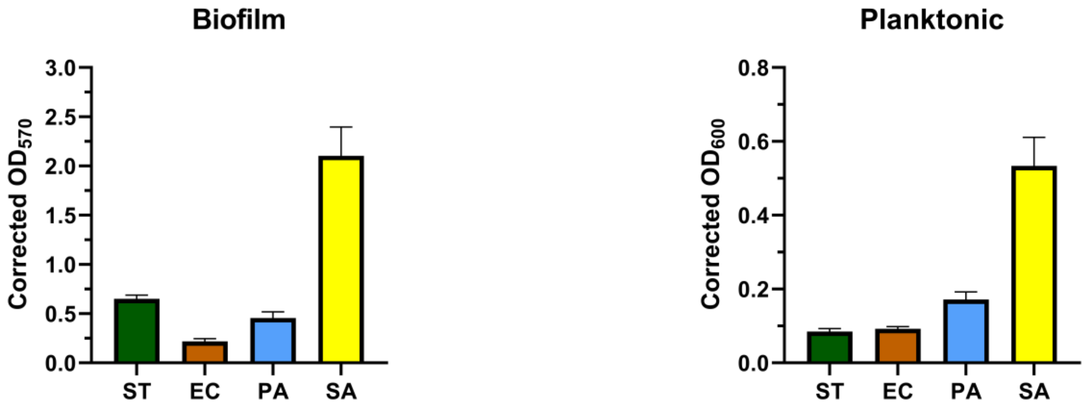

FIG S3. Relative biofilm formation and planktonic growth of the four assayed bacteria in untreated conditions, as measured in the extensive randomized validation experiment for determination of tannin activity against biofilm and planktonic bacteria. ST: *Salmonella* Typhimurium, EC: *Escherichia coli*, PA: *Pseudomonas aeruginosa*, SA: *Staphylococcus aureus*.
