# Supplementary material for: Effect of chemical modifications of tannins on their antimicrobial and antibiofilm effect against Gram-negative and Gram-positive bacteria
Source: Front Microbiol. 2023 Jan 6;13:987164. doi: 10.3389/fmicb.2022.987164 (PMC9853077; doi:10.3389/fmicb.2022.987164)
Supplement: Supplementary file 9 [file Image_7.PDF]

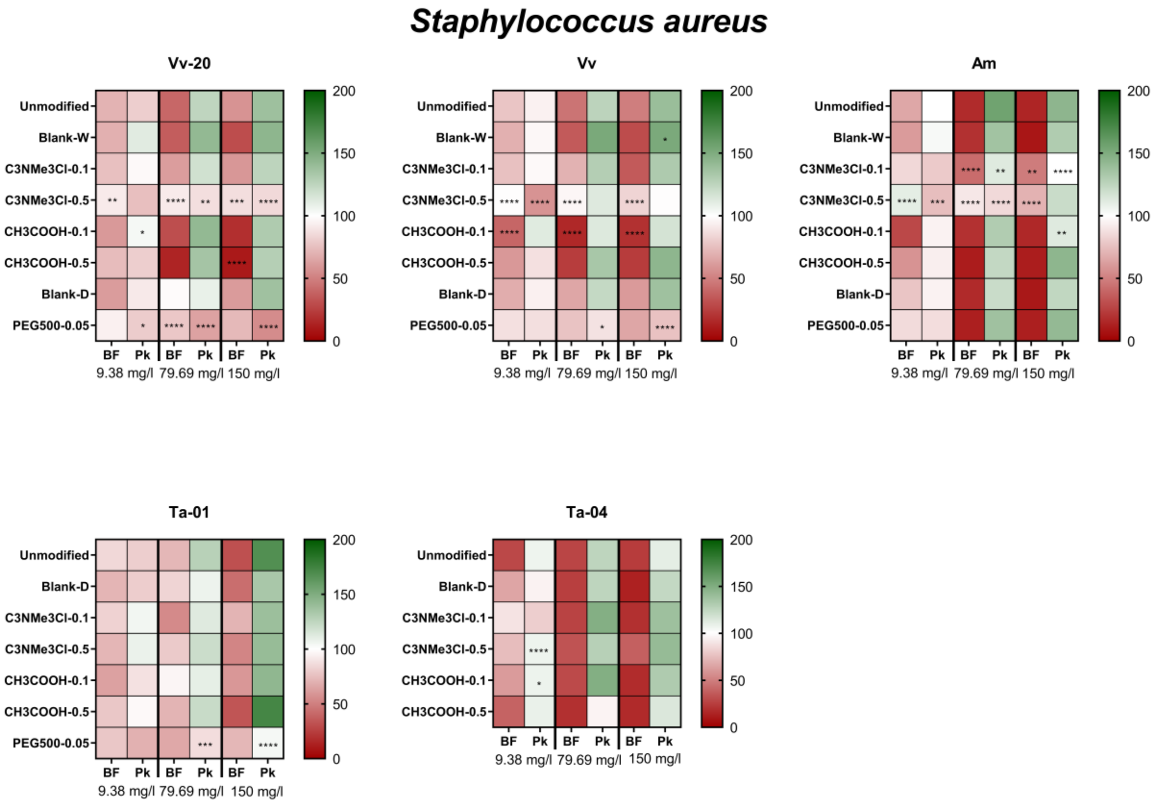

FIG S7. Effect of natural and chemically modified tannins on biofilm formation (expressed as percentage compared with positive control) and planktonic growth *Staphylococcus aureus* at 9.38 mg/l, 79.69 mg/l and 150 mg/l of tannin. The colors indicate the percentage of biofilm formation in presence of several concentrations of the assayed tannins compared to the untreated control. The asterisks indicate significant differences with the unmodified tannin, following ANOVA test with Tukey post-hoc analysis. *: $p \leq 0.05$, **: $p \leq 0.01$, ***: $p \leq 0.001$, ****: $p \leq 0.0001$.
