# Supplementary material for: Effect of chemical modifications of tannins on their antimicrobial and antibiofilm effect against Gram-negative and Gram-positive bacteria
Source: Front Microbiol. 2023 Jan 6;13:987164. doi: 10.3389/fmicb.2022.987164 (PMC9853077; doi:10.3389/fmicb.2022.987164)
Supplement: Supplementary file 10 [file Image_8.PDF]

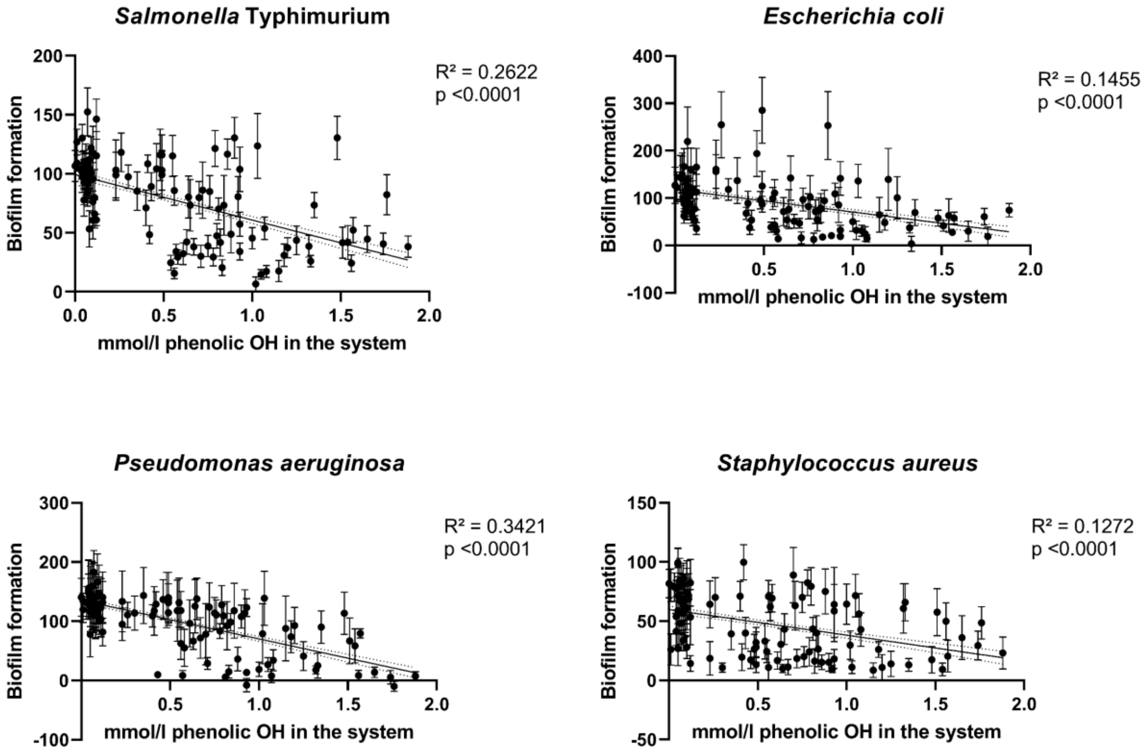

FIG S8. Correlation between the biofilm formation (measured as percentage from control) it the presence of different tannins and their respective phenolic OH content, determined via simple linear regression. Error bars represent 95% confidence interval. (A) *S.* Typhimurium (B) *E. coli* (C) *P. aeruginosa* (D) *S. aureus*.
