# Supplementary material for: Effect of chemical modifications of tannins on their antimicrobial and antibiofilm effect against Gram-negative and Gram-positive bacteria
Source: Front Microbiol. 2023 Jan 6;13:987164. doi: 10.3389/fmicb.2022.987164 (PMC9853077; doi:10.3389/fmicb.2022.987164)
Supplement: Supplementary file 11 [file Image_9.PDF]

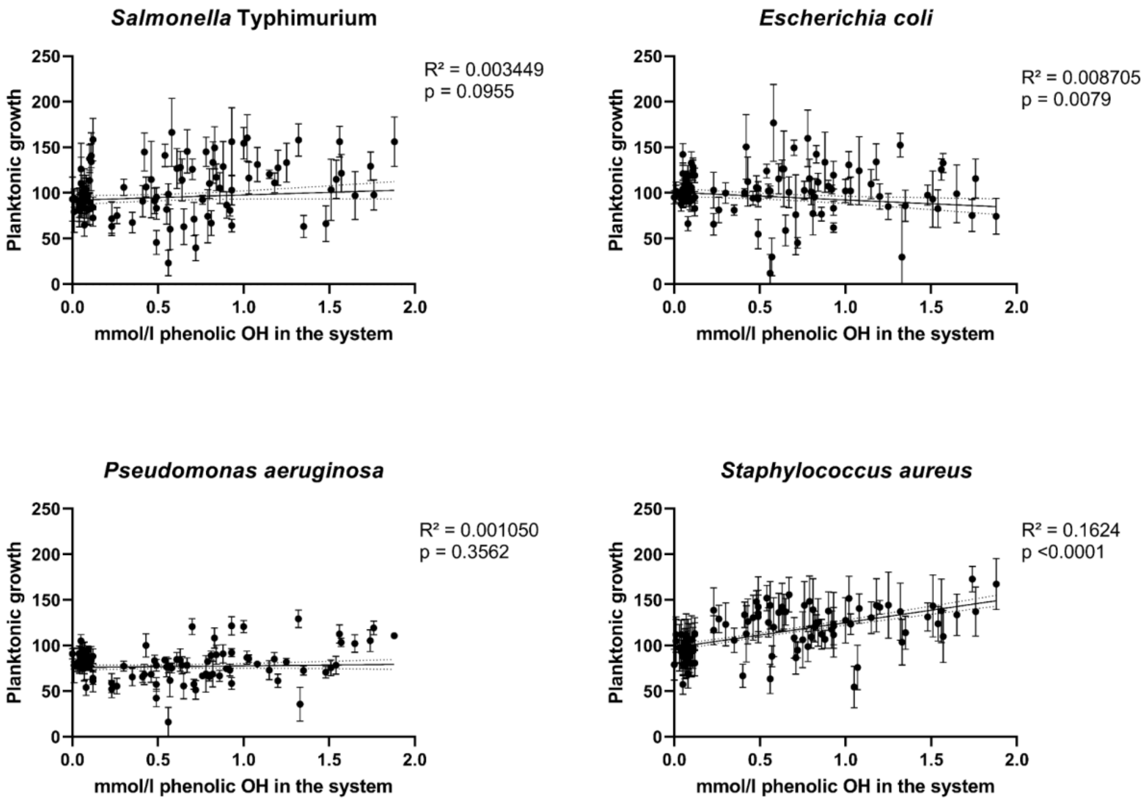

FIG S9. Correlation between the planktonic growth (measured as percentage from control) at the presence of different tannins and their respective phenolic OH content, determined via simple linear regression. Error bars represent 95% confidence interval. (A) *S.* Typhimurium (B) *E. coli* (C) *P. aeruginosa* (D) *S. aureus*.
